# Three Ships with Beriberi Outbreaks

**Published:** 1893-09

**Authors:** Albert S. Ashmead

**Affiliations:** New York, N. Y.


### Selections.

---

### THREE SHIPS WITH BERIBERI OUTBREAKS SHOWN TO HAVE HAD EXTENSIVE FORMATION OF CARBONIC OXIDES DURING THE VOYAGE.

By ALBERT S. ASHMEAD, M. D., New York, N. Y.

In *Science*, November 18, 1892, I contributed an account of the outbreak of beriberi on board the bark H. B. Cann, from Ilo-ilo, Philippine Islands, with a cargo of raw sugar. The cargo fermented during the trip, stifling fumes filled the ship, and the beriberi outbreak was considered the consequence of this state of things.

In an article, which will shortly appear in the *Medical News*, entitled Investigation of the Outbreak of Beriberi on board the bark Pax, from Ceylon, with a Cargo of Graphite, I show that from the deficient packing of 1,200 tons of graphite, the cargo was exposed to the moist air encountered on a tropical voyage, all but six of a crew of nineteen were stricken with beriberi.

The bark J. C. Warns, from Java and Macassar, with a cargo of green coffee, arrived in New York, June 23, 1890. The captain and three men had died of beriberi. The coffee had been picked and shipped too green. Mr. Tobias, consignee of the cargo, showed me a sample of it; it was charred, carbonized, and almost destroyed. The coffee had fermented. The outbreak of beriberi on a ship from Java, where the coffee has been carbonized, is a regular occurrence. Java coffee owes its value, in our market, to its color; in order to obtain this color, the captains take their cargoes quite green, which favors a slight fermentation during the trip. Sometimes they go too far; the coffee is too green, and the fermentation too violent; in such cases there is always carbonization; the grains stick together in great masses, and abundant fumes (carbonic gases) fill the ship.

The iron ship Glenmorag, Captain Currie, 133 days from Colombo, Ceylon, with 1,100 tons of graphite on board, 800 tons of cocoanut oil, etc., arrived July 17th in New York. This ship loaded in Colombo alongside the Pax (mentioned above), traveled the same course, at an interval of two weeks. She lies now at the Atlantic dock, in Brooklyn, again alongside the Pax. She had no beriberi outbreak. From her first mate I have the following information :

Crew, twenty-eight men ; captain's wife and two children on board ; or all, thirty-one. She is a Scotch Glasgow boat, and the crew is English and Scotch. Before taking this cargo, this ship had carried from Barry Dock, near Cardiff, a cargo of coal to Buenos Ayres, South America, and taken a ballast of sand to Colombo, Ceylon. Before these trips she had been in the wheat trade from Tacoma, Washington, to Havre, France. She is remarkably dry, and the cleanest ship one would wish to see. I went down her hold and examined every part of it ; there is not a smut nor a stain anywhere about it. The iron part is especially clean ; no trace of incrustation of carburetted iron, which might have indicated the action of hot, moist air on the carbon. None of the barrels containing the graphite was broken. The packing was exceedingly good ; the tonnage consisted of sticks and cocoanut hulls, so that it was impossible for the barrels to roll and break, and thus expose their contents to the action of the air.

The diet bill was about the same, or even poorer than that of the Pax. Nine casks of salted beef and seven barrels of pork were consumed during the trip. Fresh beef (tinned) three times a week, one-half pound to a man, and a half pound of salt meat on the same days ; other days a full pound of salt meat a day. One-half pound of rice for each man on Saturdays ; no vegetables, except onions, with the soup three times a week. The ship being Scotch, oatmeal made part of its fare for two and a half months after starting, when it ran out. No sickness whatever during the voyage. One death by accident. The captain attributes the condition of his cargo and his crew to the change of winds and cooler weather, which he enjoyed from the Cape of Good Hope to the North Atlantic. His log is, indeed, very different from that of the Pax.

In *Science*, Vol. XXII., No. 545, p. 16, Venable states :

The metallic carbides are usually formed by the action of intense heat upon the metal in the presence of carbon. The form of this carbon is capable of being greatly varied. Graphite, amorphous carbon, and many hydrocarbons may be used. The heat of the ordinary furnace is sufficient to form the carbides of the metals already mentioned, zinc, copper, and, notably, iron. All of these carbides, under certain conditions, give off their carbon in the form of hydrocarbons. The same smell can be detected in all during their decomposition. In some cases, as iron and zinc, the decomposition is caused by the action of an acid. The carbides of the earths (*of which graphite is one, in conjunction with iron,*) decompose in most air, and more rapidly in water.

I may point, again, here to those broken barrels of the Pax, which exposed the carbon to the influence of tropical air.

I have examined, microscopically, the blood of four of the suf ferers of the Pax, and obtained the following results :

Captain Geeseicke, sick since May 16th with beriberi ; 800 diameters, one-twelfth of an inch oil-immersion objective ; red discs, irregular in outline, congregated in masses, with ragged edges, not inclined to form rouleaux ; quite plastic ; colored streaks, or rays of pink and red, showing the presence of biliary matter, biliverdin crystals ; black spores, not free, but entangled in the hummocks of corpuscles. It may be noted that the edema of this patient's legs only left him two days before this examination.

Henry Oelrichs (second mate), German, 27 years old. Has been fourteen days sick with beriberi. Examination of the blood : 500 diameters, one-eighth of an inch objective; red corpuscles, very plastic, aggregated in hummocks. Many black spores are seen floating about, free in motion. Fibrin in excess, light in texture, and lumpy. Blood very thick, syrupy, and plastic. No motion, showing want of circulation. Excess in the coloring matter. This same case examined with 900 diameters, one-sixteenth of an inch objective immersion lens, shows excess of fibrine in ropes, biliary matter in great excess ; no crystalline formations ; blood quickly oxidizes and forms a solid mass. The black spores, above mentioned, are quickly held by the fibrin ; the red discs are distended, bladder-shaped, and have very ragged edges. The meniscus-shape is lacking, there being great irregularity in outline and color, some are even square-shaped. Some discs have an excess of color; some very pale. On the edges of the corpuscular mass the color quickly disappears, in consequence of rapid coagulation.

Isaac Hegglund, a Swede, 27 years old, has had beriberi since crossing the equator, six weeks ago. Legs are now very thin, but still some soreness remains ; knee reflexes still lost. First sound of heart prolonged. Microscopical examination of the blood, 900 diameters, one-sixteenth of an inch objective, shows rouleaux well formed, no spores, no filaments, slight feverish condition shown by spiculated outlines of some of the red corpuscles. Fibrine is assuming a normal form, showing meshes very regular ; no distension of red corpuscles.

Emil Jensen, a German, 19 years old, sixteen days sick with beriberi. Black spores in active motion and very plentiful ; freely scattered in the field of observation ; circulation very torpid ; fibrine very irregular, light in texture ; biliary matter freely scattered ; blood discs distended, and with ragged edges ; red corpuscles congregated in masses ; fibrine forming in heavy clots ; blood rapidly coagulating; black spores are quickly fastening in the fibrine.

We have here, in the fourteenth day and the sixteenth day cases, black spores in active motion and biliary matter in both cases, and the corpuscles distended, bladder-shaped, in ragged-edged condition ; the fibrine quickly clotting. And, in the captain's case, which was the worst of all, we have still black spores, biliary matter, and ragged-edged corpuscles.

In the sixth week case, a much milder case, moreover, than any of the others, it is reasonable to assume that in some way the patient has quickly eliminated the poison. There is no biliary matter in his blood, no black spores, no abnormal fibrine, no distension of red corpuscles ; the latter are perfectly formed in rouleaux.

Examination of urine of Henry Oelrichs (second mate, bark Pax), July 17, 1893 (fourteenth day of beriberi) :

Odor, light, aromatic, and feverish.
Color, light (yellow) amber.
Reaction, excessively acid.
Appearance, transparent.
Specific gravity, 1.032+.
Weight of a fluid ounce, 470.27 grains.
Solids in a fluid ounce, 35.06 grains.
Nature of deposit, mucus.
Quantity of deposit, trace.
Bile, coloring matter not present.
Salts, not present.
Sugar, Fehling's solution, trace.
Chromate solution, trace.
Nylander's solution, trace.
Sacchrimeter grammes in a liter, 0.00+.
Albumen, nitric acid, 1 fl. ℥, not present.
Picric solution, trace.
Touret's solution, trace.
Bichromate solution, not present.
Bichloride solution, trace.
Millard's solution, trace.
Polariscopic grammes in a liter, 0.00+.

Microscopical appearances :

Pus corpuscles, trace in quantity.

Epithelium, bladder, trace in quantity.

Qantitative examination :

| | |
|---|---|
| Urea, proportion per fluid ounce........ | 6.605 grains. |
| Percentage of.................... | 1.404 |
| Total, quantitative examination... | 66.050 |
| Chlorine ............................ | .960 grains. |
| | 204 |
| | 9.600 grains. |
| Sulphuric acid........................ | .992 grains. |
| | 210 |
| | 9.920 grains. |
| Phosphoric acid...................... | 1.024 grains. |
| | 201 |
| | 10.240 grains. |
| Carbonic acid gas..................... | 1.120 grains. |
| | 237 |
| | 11.200 grains. |

Results on a net basis :

| | |
|---|---|
| Urea.......................................... | 1.40 |
| Water......................................... | 95.00 |
| Sugar......................................... | 0.00+ |
| Foreign ...................................... | 2.76 |
| Albumen ...................................... | 0.00+ |
| Chlorine...................................... | .20+ |
| Sulphuric acid................................ | .21 |
| Phosphoric acid............................... | .20 |
| Carbonic acid gas............................. | .23 |
| | 100.00 |

Traces of sugar and carbonic acid gas are commonly observed in the urine of beriberi patients.

Dr. Wallace Taylor, Osaka, Japan, sends me three interesting tables, which he made from examinations of 134 cases of beriberi. These examinations were made with Hayem's hematometer and Gower's hemacytometer. The average corpuscular richness for the 134 cases is ninety-four per cent. This, he says, corresponds to the clinical experience in cases of beriberi. Most of the cases of beriberi seen by the general practitioners are well-fed, well-nourished, full-blooded appearing men. The ill-fed, poorly-nourished, weak constitution cases are the exception. During the past few years he has kept a record of the physical condition of the beriberi patients, and he gives this record, together with another record, of a beriberi hospital in Tokio:

| | Taylor. | Beriberi Hospital. | Sum. |
|---|---|---|---|
| Of strong constitution, | 323 | 593 | 916 |
| " average " | 15 | 27 | 42 |
| " weak " | 9 | 6 | 15 |

Thus, in a total of 973 beriberi patients, there were ninety-four per cent. of strong constitution, (a result almost identical with that given in his tables,) and only six per cent. of average and weak constitutions.

"These numbers," says Taylor, "are large enough to be conclusive, and anemia is not one of the pathological conditions of beriberi."

In his table No. 3 there is shown a general diminution of the hemoglobin. The average hemoglobin in 101 cases is eighty-one per cent. In some of these cases the amount is very low, being below sixty-five per cent., and with but few exceptions the per cent. of hemoglobin is below the per cent. of corpuscles, showing a deficiency of the individual corpuscles in hemoglobin.

The appearance of biliary matters, which I have shown in my analyses of the four cases of the bark Pax, would show by itself a deficiency of hemoglobin.

In the *Tribune Mèdicale*, September 10, 1891, Messrs. Bertin-Sans, and Moitessier show that it is the presence of hydrogen and carbonic acid in oxicarbonized blood that prevents the total destruction of hemoglobin.

By sweeping their solution of oxicarbonized blood and water with a current of hydrogen and carbonic acid gas, and an addition of sulphide of ammonia, they obtained the spectrum of reduced hemoglobin. They thus show that oxicarbonized hemoglobin can be readily transformed into a mixture of methemoglobin and oxide of carbon. It is, therefore, reasonable to suppose that in an outbreak of beriberi, where we have the presence of oxides of carbon and a deficiency of hemoglobin (observable in all cases of beriberi), the latter is the effect of the former.

In Japan, the universal burning of charcoal produces the oxides, which held down in the low places by the moist atmosphere of the beriberi season, there is produced on a large scale, and continually during the moist season, what happens on board of each of those ships which come to us from the East with carbonized cargoes and beriberi-sick crews.—*Science, July, 1893.*

